# Effects of partial or complete laser-assisted hatching on the hatching of mouse blastocysts and their cell numbers

**DOI:** 10.1186/1477-7827-11-21

**Published:** 2013-03-19

**Authors:** Chanchai Chailert, Usanee Sanmee, Waraporn Piromlertamorn, Sudarat Samchimchom, Teraporn Vutyavanich

**Affiliations:** 1 Division of Reproductive Medicine, Department of Obstetric and Gynecology, Faculty of Medicine, Chiang Mai University, Chiang Mai 50200, Thailand

**Keywords:** Assisted hatching, Laser, Differential staining, Zona drilling, Zona pellucida

## Abstract

**Background:**

It is still debatable whether a full-thickness assisted hatching (AH) is better than the partial zona thinning. In this research, we used a mouse model to study the effect of partial and complete laser-AH on the rate of completely hatched blastocyst and their cell numbers.

**Methods:**

In experiment 1, mouse morulae had 0, 1, 2 or 3 full-thickness openings of 10 microns created in the zona pellucida with an infrared laser beam. In the second experiment, 0, 1 and 2 openings of 20 microns were studied. In the third experiment, a full-thickness opening of 20 microns or quarter-thinning of the zonal circumference to a depth of 90% was compared with non-AH controls.

**Results:**

No difference in blastocyst formation was found in laser-treated groups and in the controls. In experiment 1, the rate of completely hatched blastocysts was significantly lower than the controls. In experiment 2 when the size of the opening was increased, blastocysts completely hatched at a significantly higher rate than that in the controls. In experiment 3, the rate of completely hatched blastocysts was the highest in the full-thickness group. Cell numbers in completely hatched blastocysts from both AH groups were significantly fewer than those in the controls.

**Conclusions:**

Full-thickness opening resulted in a higher rate of completely hatched blastocysts than quarter zonal-thinning and controls, but the cell numbers were significantly decreased.

## Background

Zona pellucida (ZP) must be shed within a time period when the uterus is receptive in order for a pregnancy to occur. Assisted hatching (AH) has been proposed to increase the hatching and embryo implantation rate, probably by enhancing early communication between the embryo and endometrium [1]. An infrared laser system appears to be the most suitable method for assisted hatching, as it has good repeatability and consistency between operators and is simple to perform [1-3].

It is debatable whether a full-thickness opening is better than the partial zona thinning. There was a concern that a hole in the zona might deprive the embryo of its protective coat against infectious or immunologic insult [3]. Blastomeres could be lost through an opening in the zona during a difficult embryo transfer or during contraction of the uterus after the transfer [3,4]. Zonal thinning was believed to be optimal in promoting complete hatching without the potential drawbacks of the full-thickness opening [5]. In the Cochrane Systematic Review [6], subgroup analysis by the extent of assisted hatching showed that there was a significant increase in the clinical pregnancy rate per woman randomized in both the zona thinning group (OR 1.33, 95% CI 1.04, 1.72) and the full-thickness AH (OR 1.23, 95% CI 1.01, 1.50). To date, there have been only one randomized trial [7] and one retrospective study [8] that compared laser zonal breaching with thinning in the human, and one study in the mouse [9], but the results were inconclusive.

Many studies showed that AH significantly increased the implantation and pregnancy rates in women with poor prognosis, such as advanced age [5,10-12], recurrent implantation failure [5,12-16], or in the frozen-thawed cycles [17-25]. However, other studies were unable to confirm the beneficial effects of AH in the general populations [26-30], or in specific groups, such as advanced age [31-37], recurrent implantation failure [21,36], or in frozen-thawed cycles [7,36,38-41]. Of more concern was that some studies reported a lower pregnancy rate after complete AH [7] or partial zona thinning [42] than that in the controls. The current opinion is that the routine use of assisted hatching for all patients is unwarranted in view of the lack of evidence of universal benefit and the potential risks [3,43].

In this research, we used a mouse model to study the effect of partial and complete AH on the rate of blastocyst formation, hatching and cell numbers. In a previous study, Montag *et al.*[44] showed that the mean numbers of embryonic cells in hatching blastocyst in the full-thickness laser AH were significantly lower than those in the non-AH controls. However, they did not report whether the decrease was predominantly in the trophectoderm or inner cell mass. Moreover, the number of cells in completely hatched blastocysts in the two groups on the same day of embryonic development was not reported. We postulated that both the complete and partial AH might have a detrimental effect on the number of cells in the hatched blastocysts.

## Methods

International Cancer Research (ICR) mice were purchased form the National Animal Institute, Mahidol University, Bangkok, Thailand. They were kept in our animal husbandry unit, at 25±2°C, under 60-70% humidity with controlled 12 hour light/12 hour dark cycles. Before the experiment, the mice were left undisturbed for five days to avoid the effects of stress from transportation. The Animal Ethics Committee of the Faculty of Medicine, Chiang Mai University approved the use of mice in our study (protocol no. 40/2553). All the procedures were performed in compliance with the EC Directive 86/609/EEC for animal experiments.

Five- to seven-week-old ICR females were super-ovulated by an intra-peritoneal injection of 10 IU of equine gonadotropin (Sigma, St. Louis, USA), followed 48 hours later by 10 IU of human chorionic gonadotropin (hCG; Pregnyl, Organon, Oss, The Netherlands). After hCG injection, females were paired with nine- to twenty-week males. They were checked for mating 16 hours later, and sacrificed by cervical vertebrae dislocation 36 hours after hCG injection. Two-cell embryos were flushed from the oviducts, and washed twice in cleavage medium (Cook, Brisbane, Australia) under paraffin oil (Medicult, Jyllinge, Denmark). They were cultured in groups of ten in 10 μL drops of cleavage medium for 48 hours without medium renewal until they became morulae.

### Interventions

The XYClone® laser system (Hamilton Thorne Biosciences, Beverly, MA, USA) was attached to an inverted microscope (Eclipse TE300, Nikon, Tokyo, Japan), equipped with a heated stage (Kitazato, Fujinomiya, Japan). The machine emitted an infrared laser beam with a wavelength of 1.48 μm, at a power of 140 mW. The pulse was set to create a 10-μm hole in the mouse ZP.

Up to five morulae were placed in a 10 μL drop of medium under oil and put on the microscopic stage. The laser beam was fired once at the ZP to create a full-thickness opening of 10 μm, or twice in adjacent area for an opening of 20 μm. For ZP thinning, the same setting of laser was used to fire in a consecutive area along the periphery of zona to leave a thin rim (~ 10%) of the original thickness, covering an area of 1/4 of the ZP circumference. Control morulae were placed on the stage for the same duration as the experimental groups, but laser drilling was not performed.

In experiment 1, four hundred and forty mouse morulae were divided into a control and three laser-AH groups (1 hole, 2 holes or 3 holes evenly apart, each of 10 μm in size) (Table 1).

**Table 1:** Summary of embryos utilized in this study

**Experiment 1**	**Experiment 2**	**Experiment 3**
**Total = 440**	**Total = 360**	**Total = 540**
Control = 110	Control = 120	Control = 180
1-hole (10 μm ) = 110	1-hole (20 μm ) = 120	Complete AH = 180
2-holes (10 μm ) = 110	2-holes (20 μm ) = 120	Zona thinning = 180
3-holes (10 μm ) = 110		

In experiment 2, three hundred and sixty morulae were divided into a control and two laser-AH groups (1 or 2 holes at opposite pole, each measured 20 μm in size).

In experiment 3, five hundred and forty morulae were divided into a control and two laser-AH groups (full-thickness opening of 20 μm or quarter-thinning of the zonal circumference to a depth of 90%).

### Culture condition and assessment of embryo development

After the intervention, groups of ten morulae were cultured together in 10 μL drops of blastocyst medium (Cook, Brisbane, Australia) under paraffin oil, in an atmosphere of 6% CO_2_, 5% O_2_ and 89% N_2_ at 37°C. Embryos were cultured in this medium for up to 72 hours without medium renewal. Embryo development was observed at 24, 48 and 72 hours after AH.

### Differential staining of inner cell mass (ICM) and trophectoderm (TE) cells

Differential staining was performed on all completely hatched blastocysts, using the protocol described by Pampfer *et al.*[45]. In brief, the blastocysts were washed three to four times in calcium- and magnesium-free buffer, before exposure to rabbit anti-mouse antibody (Sigma M 5774; concentration 1:50) for 30 minutes at 37°C. After washing, they were transferred into guinea pig complement serum (Sigma S 1639) with propidium iodide (Sigma P 4170) and bisbenzimide (Sigma B 2261) at 37°C for 10–15 minutes. The blastocysts were washed and transferred onto glass slides to allow air dry. The slides were mounted in glycerol, and the number of ICM and TE cells were counted under a Nikon E600 epifluorescence microscope, equipped with LUCIA FISH program (Laboratory Imaging, Prague, Czech Republic).

### Statistical analysis

The STATA program version 8.2 (College Station, Texas, USA) was used to perform Chi-square tests to compare the proportions of morulae that became blastocysts, and proportions of hatching and completely hatched blastocysts in the experimental and control groups at 24, 48, and 72 hours after AH, respectively. Fisher exact test was used when any of the expected cell frequencies was <5. The mean numbers of inner cell mass (ICM) and trophectoderm (TE) cells of hatched blastocysts from different groups were compared by one-way ANOVA, with Scheffe post-hoc tests as appropriate. A two-tailed *P*<0.05 was considered statistically significant. Bonferroni correction was used as a safeguard against multiple tests of statistical significance on the same data by dividing the cut-off *P*-value of <0.05 by the number of tests done on that data set.

## Results

A total of 1340 morulae were included in the study. In experiment 1, the proportions of morulae that became blastocysts 24 hours after the interventions in the control (108/110) and 3 laser AH groups (109/110, 109/110 and 110/110 for the 1-, 2- and 3-hole groups, respectively) were not statistically different (*P*=0.905). The proportions of hatching blastocysts at 48 hours were significantly higher in the AH groups (94/109, 99/109 and 102/110, respectively) than controls (51/108; *P*=0.000). There was a higher hatching rate when the number of holes was increased. In contrast, the rate of completely hatched blastocysts at 72 hours was significantly higher in the control (44/108) than the AH groups (19/109, 14/109 and 12/110, respectively; *P*=0.000). A higher proportion of blastocysts were entrapped when the number of holes was increased. There was a significant difference in the number of ICM, TE and total cells, but not in ICM/TE ratio between AH and non-AH blastocysts (Table 2).

**Table 2:** Number of cells in the inner cell mass (ICM), trophectoderm (TE), total cells, and ICM:TE ratio of hatched blastocysts in the control and laser groups in experiment 1

	**Control**	**1-hole**	**2-hole**	**3-hole**	**P**
	**(n=44)**	**(n=19)**	**(n=14)**	**(n=12)**	
ICM	23.6±4.6	19.5±3.8	16.5±3.0	15.2±2.3	0.000^**a**^
TE	68.7±15.9	59.7±14.1	53.1±8.4	50.3±9.8	0.000^**b**^
ICM/TE	0.4±0.1	0.3±0.1	0.3±0.1	0.3±0.1	0.184
Total	92.2±18.3	79.2±16.4	68.9±9.4	65.5±10.1	0.000^**c**^

Values are mean ± standard deviation (SD).

One-way ANOVA with Scheffe post-hoc test when P<0.0125 (P<0.05/4).

^a^ control vs. 1-hole group (*P*=0.004), control vs. 2-hole group (*P*=0.000), control vs. 3-hole group (*P*=0.000), and 1-hole vs. 3-hole group (*P*=0.039).

^b^ control vs. 2-hole group (*P*=0.006), and control vs. 3-hole group (*P*=0.002).

^c^ control vs. 1-hole group (*P*=0.036), control vs. 2-hole group (*P*=0.000), and control vs. 3-hole group (*P*=0.000).

In experiment 2, the proportions of morulae that became blastocysts in the controls (118/120) and 2 laser AH groups (119/120 and 118/120 in the 1- and 2-hole groups, respectively) were not different (*P*=1.000). The proportion of completely hatched blastocysts was significantly higher in both AH groups (66/119 and 63/118, respectively) than that in the controls (44/118; *P*=0.009). There was a significant difference in the number of ICM, TE, and total cells between control and laser-AH blastocysts, but not in ICM/TE ratio (Table 3).

**Table 3:** Number of cells in the inner cell mass (ICM), trophectoderm (TE), total cells, and ICM:TE ratio of hatched blastocysts in the control and laser groups in experiment 2

	**Control**	**1-hole**	**2-hole**	***P***
	**(n=44)**	**(n=66)**	**(n=63)**	
ICM	23.5±2.7	20.7±3.1	19.6±3.5	0.000^**a**^
TE	64.8±6.8	55.7±9.4	53.7±10.6	0.000^**b**^
ICM:TE	0.4±0.04	0.4±0.05	0.4±0.06	0.661
Total	88.2±8.1	76.3±11.6	73.1±12.9	0.000^**c**^

Values are means ± standard deviation (SD).

One-way ANOVA with Scheffe post-hoc test when P<0.0125 (P<0.05/4).

^**a**^ control vs. 1-hole group (*P*=0.000), and control vs. 2-hole group (*P*=0.000).

^**b**^ control vs. 1-hole group (*P*=0.000), and control vs. 2-hole group (*P*=0.000).

^**c**^ control vs. 1-hole group (*P*=0.000), and control vs. 2-hole group (*P*=0.000).

In experiment 3, the proportions of morulae that became blastocysts were not different between control and the two laser-AH groups (Table 4). After 48 hours, the proportions of hatching blastocysts in the two laser-AH groups were higher than that in the control. The proportion of completely hatched blastocyst was highest in the full-thickness AH group, compared to those in the control and quarter laser-thinning group. The rate of blastocyst trapping was likewise lowest in the full-thickness AH group. There was a significant difference in the number of ICM, TE, and total cells in both AH groups when compared with controls (Table 5).

**Table 4:** Effects of laser quarter-thinning and full-thickness opening on the rate of blastulation, blastocyst hatching, and completely hatched blastocyst at 24, 48 and 72 hours post assisted hatching, compared with non-manipulated controls in experiment 3

	**Control**	**Full-thickness**	**Thinning**	***P***
	**(n=180)**	**(n=180)**	**(n=180)**	
Blastulation rate	176 (97.8%)	173 (96.1%)	178 (98.9%)	0.260^**a**^
Hatching rate	97 (55.1%)	167 (96.5%)	167 (93.8%)	0.000^**b**^
Hatched rate	75 (42.6%)	106 (61.3%)	69 (38.8%)	0.000^**c**^

^**a**^ Fisher’s Exact Test = 2.815.

^**b**^ Pearson Chi-Square test, χ ^2^ = 126.59.

^**c**^ Pearson Chi-Square test, χ ^2^ = 20.292.

**Table 5:** Number of cells in the inner cell mass (ICM), trophectoderm (TE), ICM:TE ratio and total cell number in control, full-thickness and quarter zonal thinning groups in experiment 3

	**Control **^**a**^	**Full-thickness **^**a**^	**Thinning**	***P ***^**b**^
	**(n=62)**	**n=99)**	**(n=69)**	
ICM	23.7±3.4	20.85±3.08	21.63±3.06	0.000 ^**c**^
TE	65.9±9.9	55.19±10.19	58.76±8.67	0.000 ^**d**^
ICM:TE	0.36±0.04	0.38±0.05	0.36±0.03	0.02
Total	89.4±12.7	76.0±12.8	80.4±11.3	0.000 ^**e**^

Values are mean ± standard deviation (SD).

^**a**^ Some blastocysts were excluded due to accidental lost or poor staining.

^**b**^ As a safeguard against multiple tests of statistical significance on the same data, the Bonferroni correction was used to adjust the *P*-value. In this case, a *P*-value <0.05/4 or <0.0125 was considered to indicate a significant difference.

One-way ANOVA with Scheffe post-hoc test when *P*<0.0125.

^**c**^ control vs. full-thickness group (*P*=0.000), and control vs. quarter-thinning group (*P*=0.001), and full-thickness vs. quarter thinning group (*P*=0.307).

^**d**^ control vs. full-thickness group (*P*=0.000), control vs. quarter thinning group (*P*=0.000), full-thickness vs. quarter-thinning group (*P*=0.076).

^**e**^ control vs. full-thickness group (*P*=0.000), control vs. quarter thinning group (*P*=0.000) full-thickness vs. quarter thinning group (*P*=0.09).

## Discussion

In the first experiment, we confirmed previous studies that a small opening in the zona, although associated with early hatching, ended up with a higher rate of blastocyst entrapment [19,46-48]. The entrapment increased with increasing numbers of openings. In experiment 2, laser AH of adequate size was associated with not only early but also a higher rate of complete blastocyst hatching. In this case, two opposite openings did not result in an increase entrapment. Cohen and Feldberg [48] clearly showed that embryos with multiple openings in their zona preferentially hatched through the largest one. Both their study and ours indicated that the size was more important than the number of zonal opening.

Several previous studies supported the practice of zona thinning rather than total breaching [8,46]. Zonal thinning was believed to be optimal in promoting complete hatching without the potential drawbacks of the full-thickness opening. Moreover, increasing the area of the zona thinning might encompass the site of natural hatching. In our study, we chose to perform quarter zonal thinning rather than thinning of half of the ZP circumference. The reason was that it would save time as well as decrease the number of laser shots. The embryos would be outside the incubator shorter and the risk of temperature increase in the immediate vicinity of the embryos from laser thermal shock would be minimized. It was disappointing that quarter laser thinning of the ZP did not increase the percentage of completely hatched blastocysts. On the contrary, it even increased the chance of trapping. Our result was consistent with that reported by Tinney *et al*. [49]. They performed zonal thinning through seven consecutive shots in the zona, and found an increase in the rate of incompletely hatched blastocysts. Interestingly, a randomized trial of laser quarter zona thinning by Valojerdi *et al*. [42] showed a significant decrease in clinical pregnancy and implantation rates in vitrified/warmed human embryos, compared with non-hatched controls.

In this study we performed differential counts of ICM and TE cells in all completely hatched blastocysts. The mean numbers of embryonic cells (ICM+TE) in our study were higher than those reported by Montag *et al*. [44] and Fathi *et al*. [50]. This was because they counted cells in hatching [44] or expanding [50] blastocysts rather than completely hatched blastocysts. Our study and that by Montag *et al*. [44] agreed that blastocyst cell counts significantly decreased after laser AH, when compared with the control. Fathi *et al*. [50] also observed a similar decrease in the number of cells in the ICM, TE and total cells, but the difference only reached statistical significant in the case of the ICM. In our study, we found that the decrease occurred proportionately in the ICM and TE cells, with no change in the ICM/TE ratio. The decrease was also observed in the case of partial zona thinning, albeit to a lesser degree than that seen in complete AH. Montag *et al*. [44] reasoned that the presence of a laser-drilled opening in the zona allowed hatching to occur through the opening as soon as the blastocyst started to expand. In contrast, in the untreated control, hatching was delayed until sufficient number of embryonic cells was available to overcome the resistance of the zona. However, this assumption alone could not explain why the number of cells in the completely hatched blastocysts in the AH group was significantly fewer than controls on the same day of embryonic development. Perhaps, an alternative explanation could be that blastocyst expansion stimulated the proliferation of cells in the blastocysts. Without blastocyst expansion in the case of full-thickness AH, or with partial expansion in zonal thinning, the number of cells in the resulting blastocysts decreased in a dose–response manner. Given this assumption, one could explain why the routine use of AH in normal embryos might not be beneficial or even be detrimental. On the other hand, AH may be useful in cases with real zona hardening, despite the lower number of cells in the resulting blastocysts.

The limitation of our study was that we did not transfer the laser-treated embryos into the uterus, and hence implantation and pregnancy rates could not be determined. Moreover, the hatching process *in vitro* and *in vivo* might be different. There could also be species difference, and one should be very cautious in projecting data from the mouse directly to the human.

Although the use of an *in vitro* mouse model was our weakness, it was also our strength. We were able to study a large numbers of embryos, which was not possible with humans due to ethical reasons. Our *in vitro* study allowed us to follow the fate of all embryos to the end. The percentage of trapped and completely hatched blastocysts could be accounted for, and the number of ICM and TE cells could be enumerated in all completely hatched blastocysts.

## Conclusions

In conclusion, the size of the zonal opening was more important than its number. A full-thickness opening allowed more complete blastocyst hatching than quarter zona thinning. The numbers of cells in the ICM and TE of hatched blastocysts after full-thickness or partial AH were lower than those in the non-manipulated controls, but there was no significant change in the ICM to TE ratio. The blastocyst cell number was slightly lower in the complete than the partial AH group, but the difference was not statistically significant.

## Abbreviations

AH: Assisted hatching; hCG: Human chorionic gonadotropin; ICM: Inner cell mass; ICR: International Cancer Research; TE: Trophectoderm; μL: Microliter; μm: Micron; ZP: Zona pellucida.

## Competing interests

The authors declare that they have no competing interests.

## Authors’ contributions

CC participated in the design, carried out experiments 1–3, and helped in data analysis and revising the manuscript critically for important intellectual content. US and SS helped CC in carrying out experiments 1–3, read, revised and approved the final manuscript. WP participated in the design and coordination of the study, took part in the analysis, interpretation revised and approved the manuscript. TV made substantial contributions to conception, design, analysis and interpretation of the data, and wrote the manuscript. All authors read and approved the final manuscript.
